# Triose Phosphate Isomerase Deficiency Is Caused by Altered Dimerization–Not Catalytic Inactivity–of the Mutant Enzymes

**DOI:** 10.1371/journal.pone.0000030

**Published:** 2006-12-20

**Authors:** Markus Ralser, Gino Heeren, Michael Breitenbach, Hans Lehrach, Sylvia Krobitsch

**Affiliations:** 1 Max Planck Institute for Molecular Genetics Berlin, Germany; 2 Department of Cell Biology, University of Salzburg Salzburg, Austria; Institut Pasteur, France

## Abstract

Triosephosphate isomerase (TPI) deficiency is an autosomal recessive disorder caused by various mutations in the gene encoding the key glycolytic enzyme TPI. A drastic decrease in TPI activity and an increased level of its substrate, dihydroxyacetone phosphate, have been measured in unpurified cell extracts of affected individuals. These observations allowed concluding that the different mutations in the TPI alleles result in catalytically inactive enzymes. However, despite a high occurrence of TPI null alleles within several human populations, the frequency of this disorder is exceptionally rare. In order to address this apparent discrepancy, we generated a yeast model allowing us to perform comparative *in vivo* analyses of the enzymatic and functional properties of the different enzyme variants. We discovered that the majority of these variants exhibit no reduced catalytic activity per se. Instead, we observed, the dimerization behavior of TPI is influenced by the particular mutations investigated, and by the use of a potential alternative translation initiation site in the TPI gene. Additionally, we demonstrated that the overexpression of the most frequent TPI variant, Glu104Asp, which displays altered dimerization features, results in diminished endogenous TPI levels in mammalian cells. Thus, our results reveal that enzyme deregulation attributable to aberrant dimerization of TPI, rather than direct catalytic inactivation of the enzyme, underlies the pathogenesis of TPI deficiency. Finally, we discovered that yeast cells expressing a TPI variant exhibiting reduced catalytic activity are more resistant against oxidative stress caused by the thiol-oxidizing reagent diamide. This observed advantage might serve to explain the high allelic frequency of TPI null alleles detected among human populations.

## Introduction

Triosephosphate isomerase deficiency has been initially described in 1965 [1]. It is a unique glycolytic enzymopathy with autosomal recessive inheritance that is characterized by chronic haemolytic anaemia, cardiomyopathy, susceptibility to infections, severe neurological dysfunction, and, in most cases, death in early childhood [2]. Thirteen different mutations in the respective gene, which is located at chromosome 12p13 and encodes the ubiquitous housekeeping enzyme triosephosphate isomerase (TPI), have been discovered so far [2], [3]. TPI is a crucial glycolytic enzyme and catalyzes the interconversion of dihydroxyacetone phosphate (DHAP) and glyceraldehyde-3-phosphate. A marked decrease in TPI activity and an accumulation of DHAP have been detected in erythrocyte extracts of homozygous and compound heterozygous TPI deficiency patients. Remarkably, heterozygous individuals are clinically unaffected, even if their residual TPI activity is reduced to about 50% compared to normal activity [2], [4], [5]. Moreover, the frequency of heterozygous unaffected individuals in all human populations investigated is significantly higher than expected from the rare incidence of homozygous or compound heterozygous TPI deficiency patients [5]–[9]. Interestingly, mice studies demonstrated that mutations resulting in catalytically inactive TPI variants (null alleles) led to early prenatal lethality in the homozygous state, an incidence that might also arise in humans [7], [10]–[12].

Bioinformatic analyses have predicted that the human pathogenic mutations, which are not restricted to a specific domain or region within the enzyme, could affect the substrate binding site or the dimerization interface of TPI [2], [13]. The most prominent missense mutation detected in TPI deficiency patients occurs at codon 104 in the *TPI* gene encoding aspartic acid instead of glutamic acid within the enzyme (Glu104Asp variant) and accounts for approximately 80% of mutant alleles within Northern European kindreds with clinical TPI deficiency [14], [15]. Remarkably, this variant is the only one observed to be homozygous among TPI deficiency patients. Other amino acid exchanges in the TPI protein, such as Cys41Tyr and Ile170Val, have been predicted to interfere with both the substrate binding and the dimerization site possibly affecting the catalytic activity plus molecular stability of TPI; both pathogenic TPI variants have been identified in unrelated European kindreds [2], [13], [15]. Furthermore, other missense mutations like a mutation at codon 240 in the *TPI* gene encoding leucine instead of phenylalanine within the enzyme (Phe240Leu variant) could have an effect on the substrate binding site. Other mutations, for instance, the pathogenic TPI variants Gly122Arg or Val154Met could not be assigned to defined domains. However, Perry and Mohrenweiser showed that the Gly122Arg TPI variant, which was identified as an electromorphic variant by screening 3,400 persons in a Caucasian population, is a thermolabile enzyme possibly indicating improper folding [14], [16]. Furthermore, a start codon mutation has been identified in a French family (Met1_AAG mutation, TPI Paris) as well as a frame shift mutation at codon 28 or mutations within the upstream region of the *TPI* gene [3], [17].

To date, the best-studied family affected with TPI deficiency is a Hungarian family in which two germ-line identical compound heterozygote brothers have inherited a missense mutation at codon 240 encoding the Phe240Leu TPI variant and a nonsense mutation at codon 145 (Glu145TER) leading to a truncated TPI protein [18], [19]. Interestingly, these brothers are suffering from an atypical moderate form of TPI deficiency, although both have an extremely reduced activity of TPI with less than 5% of normal enzyme activity and a particularly high level of cellular DHAP. Strikingly, only one of the brothers has developed neurological symptoms indicating that the two mutations alone cannot explain the variance in clinical symptoms [18], [19]. In order to gain insight into the molecular processes causing the different phenotypes of the two brothers, studies have been performed that demonstrate variations in the levels of antioxidants [20], in lymphocyte TPI activity [19], in red blood cell membrane fluidity and enzyme activities and in the molecular composition of phospholipid subclasses between the two brothers [21], [22]. Moreover, it has been discovered that the Phe240Leu TPI variant binds with higher affinity to a yet unidentified component of the red blood cell membrane and to microtubules in comparison to wild-type TPI, leading to the assumption that this process could contribute to the extremely low TPI activity in the disease state [23].

According to the above-mentioned data, it is quite obvious that further functional analysis of the different TPI variants is mandatory for comprehending the molecular mechanisms underlying pathogenesis in TPI deficiency.

## Methods

### Plasmid construction and mutagenesis

We used the cDNA clones pOTB7-MGC14348 (IRALp962K2120 (RZPD); accession number BC007812) and plasmid pACT2-NTint5, which was isolated from a human fetal brain cDNA library (Clontech) for the generation of the different TPI expression plasmids described in this study. Constructs encoding the TPI variants TPI Met1_AAG (TPI Paris) or TPI Phe240Leu, respectively, were produced by PCR using oligonucleotides introducing the respective mutation as indicated in the Supporting Information. To construct expression plasmids encoding the TPI variants Cys41Tyr, Glu104Asp, Gly122Arg and Ile170Val, the diverse mutations were introduced via PCR using the respective oligonucleotide pairs, which overlap in 5′ to 3′ direction as well as in 3′ to 5′ direction. Then, the two resultant PCR fragments were utilized as DNA templates for a subsequent PCR applying oligonucleotides designed for the amplification of the full coding sequence of *TPI* (please refer to the Supporting Information for oligonucleotide sequences). Afterwards, the DNA fragments were purified and subcloned into the *Bam*HI and *Xho*I sites of the centromeric expression vector p416GPD (GeneBank accession number DQ269148, [24]) or into the *Bam*HI and *Xho*I sites of the high-copy expression vector p426GPD, respectively (DQ019861, [24]).

For the directed yeast two-hybrid analysis, we generated the respective bait and prey constructs encoding the various LexA-TPI or AD-TPI fusion proteins by ligating the different DNA fragments amplified by PCR into the *Sal*I and *Not*I sites of the bait plasmid pBTM117c or the prey plasmid pACT4-1b.

To create the mammalian expression plasmids encoding an N-terminal fragment of human TPI fused to the green fluorescent protein (GFP), the first 200 bp of the *TPI* coding sequences were amplified via PCR using the oligonucleotide pair TPI-pEGFPN1-s-Sal1 and TPI-pEGFPN1-as-BamH1, TPI_Met1_AAG_-pEGFPN1-s-Sal1 and TPI-pEGFPN1-as-BamH1 for introducing a mutation at the start codon or TPI_Ser3_TER_-pEGFPN1-s-Sal1 and TPI-pEGFPN1-as-BamH1 for introducing an artificial stop codon. Subsequently, the PCR fragments were purified and subcloned into the *Sal*I and *Bam*HI sites of the expression vector pEGFP-N1 (Clontech, U55762).

The mammalian expression plasmids pTL-Flag-TPI-WT and pTL-Flag-TPI-Glu104Asp were generated by subcloning the corresponding DNA fragments from pACT-TPI-WT or pACT-TPI-Glu104Asp into the *Xho*I/*Not*I sites of the vector pTL-FlagC, respectively. PCR was performed under conditions described earlier [25] and all resultant products were verified by sequencing.

### Yeast strains and cultivation

Wild-type strains BY4741 (MAT*a his3*Δ*1 leu2*Δ*0 met15*Δ*0 ura3*Δ*0*) [26] and L40ccua (MAT*a his3*Δ*200 trp1–901 leu2–3,11 gal8 canR cyh2R*) [27] were grown in rich medium (YPD) supplemented with 2% glucose. For the generation of the Δ*tpi1* strain MR100 (MAT*a his3*Δ*1 leu2*Δ*0 met15*Δ*0 ura3*Δ*0 LEU2::tpi1*) the *LEU2* coding sequence was amplified via PCR using the plasmid pACT2 (Clontech) as DNA template and the oligonucleotides *LEU2*::*tpi*-s and *LEU2*::*tpi*-as that each encompasses a 35 nucleotide region homologous to the *TPI1* coding sequence. After transformation of BY4741 cells via the lithium acetate method [28], transformants were selected on SC^-leu^ medium (synthetic complete medium without leucine) supplemented with 3% (v/v) ethanol/0.1% (v/v) glucose, because yeast cells deficient for the Tpi1 enzyme are unable to grow on medium solely supplemented with glucose as carbon source [29]–[31]. To verify the gene replacement of the *TPI1* coding sequence, yeast clones exhibiting no growth on media supplemented with glucose as carbon source have been selected and the correct insertion of the DNA fragment was confirmed by PCR and by western blot analysis using a polyclonal TPI serum (1∶4000, [32]). The preparation of yeast genomic DNA, ethanol lysates, SDS-PAGE and immunoblotting were performed using standard protocols as described earlier [33], [34]. To further exclude a second integration of the *LEU2* marker gene in the genome of the generated *Δtpi1* strain, the wild-type yeast *TPI1* gene was recombined back into the *TPI1* locus and transformants were tested for the loss of leucine prototrophy. One *Δtpi1* yeast clone that has been verified by this set of experiments was named MR100 and used for our experimental approaches. If not indicated otherwise, all *Δtpi1* cells were grown in media supplemented with 3% ethanol and 0.1% glucose.

For the complementation analyses, we transformed the *Δtpi1* strain MR100 with centromeric plasmids for the expression of the human TPI variants (Met1_AAG, Cys41Tyr, Glu104Asp, Gly122Arg, Ile170Val and Phe240Leu) as indicated and selected single colonies on SC^-leu-ura^ synthetic media supplemented with 3% ethanol/0.1% glucose. Then, the obtained transformants were transferred onto media plates containing glucose as carbon source allowing only yeast cells with functional TPI to grow as well as onto plates containing 3% ethanol/0.1% glucose as control. For this analysis, centromeric plasmids were used for cloning purposes, since these plasmids are known to be highly stable in yeast and are generally present as a single copy per yeast cell [24]. To prevent unwanted regulatory effects in our analyses, the constitutive *GPD* promoter was chosen for the expression of exogenous TPI. Additionally, to evaluate the biological reliability of our system, we expressed yeast Tpi1 from a centromeric plasmid in the strain MR100 as well. Enzymatic Tpi activity was determined as described below and compared to the enzyme activity of wild-type yeast strain BY4741. No significant differences in Tpi activity were observed in case the Tpi1 enzyme was expressed from the centromeric plasmid or from the genome (data not shown).

Resistance of *Δtpi1* yeast expressing wild-type or the different pathogenic TPI variants to oxidants was analyzed as described earlier [35]. Briefly, the respective yeast cultures were grown to stationary phase and serially diluted to OD_600_ values of 3.0, 1.0, 0.3 and 0.1. Then, 10 µl aliquots of each culture were spotted onto SC plates containing various concentrations of hydrogen peroxide (1–4 mM; in 0.5 mM steps) tert-butyl hydroperoxide (0.6–2 mM; 0.2 mM steps), cumene hydroperoxide (0.05–0.25 mM; 0.05 mM steps) and diamide (1.2–1.9 mM; 0.1 mM steps). Sensitivity was determined by comparing the growth between the different strains after plates were incubated for 3 days at 28°C. Aberrant enrichment of reactive oxygen species was determined by dihydroethidium staining of yeast as described earlier [35].

### Directed yeast two-hybrid analysis

To investigate the dimerization behavior of the different pathogenic TPI variants, we exploited the yeast two-hybrid system. We generated a number of bait and prey plasmids as described above and in the Supporting Information. These plasmids were used to transform the yeast strain L40ccua to express the different bait and prey proteins in various combinations as indicated in the corresponding figures. Transformants were selected on SC media lacking tryptophan and leucine. To analyze the activity of the reporter genes, yeast clones were spotted onto SC medium lacking tryptophan, leucine, histidine and uracil. Growth of yeast on selective media was analyzed after incubation of plates for 48–72 h at 30°C. The *lacZ* reporter gene activity was analyzed using an assay based on o-nitrophenyl-β-D-galactopyranoside (Applichem) as described earlier [36]. For the detection of the bait and prey proteins in the immunoblot analysis, the Gal4AD antibody (Sigma, 1∶5000) or the polyclonal lexA serum ([36], 1∶5000) was used.

### TPI activity assay

To measure the catalytic activity of the human TPI variants in yeast, an enzyme-coupled assay based on a protocol developed by Maitra and Lobo was performed [37]. Briefly, the same numbers of yeast cells from logarithmically grown cultures were collected. Then, the cells were washed and frozen at −80°C. After thawing, the cells were resuspended in PBS, pH 7.4 containing a protease inhibitor cocktail (“Complete”, Roche) and lysed using glass beads (425–600 µm, Sigma). The total protein concentration for each yeast lysate was determined using a Bradford assay (Biorad). Then, equal protein concentrations were added to PBS buffer containing 1 U glycerol-3-phosphate dehydrogenase and 0.3 mM NADH (Sigma). Subsequently, the enzymatic reaction was started by addition of 10 µl 40 mM glyceraldehyde-3-phosphate (Sigma). The oxidation of NADH was followed measuring the OD_340_ in 5 s intervals for 3 min using a spectrophotometer (Amersham Ultrospec). Afterwards, the enzymatic activity of TPI under steady-state conditions was calculated as average substrate conversion per minute and the activity of the pathogenic TPI variants was compared to the activity of the wild-type enzyme.

### Mammalian cell culture and transfection

COS1 cells were grown in Dulbecco's modified Eagle medium (DMEM, Gibco) supplemented with 5% fetal bovine serum, 50 U/ml penicillin and 50 µg/ml streptomycin (Biochrom) in a humidified 5% CO_2_ atmosphere at 37°C. Transfection was performed using Polyfect (Qiagen) as recommended by the manufacturer. To prepare cell lysates, the cells were washed in PBS and lysed in a buffer composed of 50 mM Tris-HCl, 100 mM NaCl, 5 mM MgCl_2_, 0.5% NP40, 1 mM EDTA, pH 8.8, 25 U/ml benzonase (Merck) and 2.5% protease inhibitors (“complete”, Roche). SDS-PAGE and immunoblotting were performed as described [34]. GFP fusion proteins were detected using a monoclonal GFP antibody (Roche, 1∶2000) and FLAG-tagged fusion proteins were visualized by using a polyclonal antibody directed against the FLAG tag (Sigma, 1∶5000). Tubulin was detected using a monoclonal antibody (Sigma, 1∶2000) and endogenous TPI was detected by using a polyclonal serum ([32], 1∶5000).

### Structural visualization

TPI structural coordinates were obtained from Kinoshita et al (MMDB: 32814, [38] ) and visualized using the Cn3D software package (NCBI), version 4.1.

## Results

### Human wild-type and pathogenic TPI variants complement the loss of yeast Tpi1 *in vivo*

**Figure 1:**
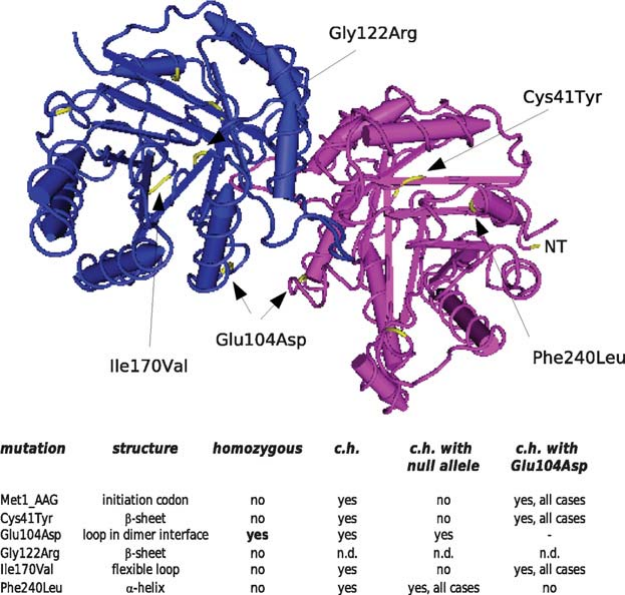
Structural model of human TPI. (Upper panel) The pathogenic TPI variants Cys41Tyr, Glu104Asp, Gly122Arg, Ile170Val and Phe240Leu were assigned to the crystal structure of human TPI generated by Kinoshita et al [38]; NT: amino terminus. **(Lower panel)** Appearance of pathogenic TPI variants among TPI deficiency patients (c.h.: compound heterozygous; n.d.: not determined).

Structure function relationship investigations have predicted that the mutations identified to be responsible for TPI deficiency may affect the tertiary or quaternary structure of TPI, hence, resulting in a strong reduction in the catalytic activity of the enzyme as determined within affected individuals; however, they are not restricted to a specific domain or region within the enzyme ([2], [13], [14] and Fig. 1). Nevertheless, patient's metabolites do highly depend on the genetic background and environmental factors. Therefore, a model system allowing study of the enzymatic and functional properties of wild-type and pathogenic TPI variants without additional side effects due to differing environment and genetic background is required to gaining insights into the molecular mechanism implicated in TPI deficiency.

**Figure 2:**
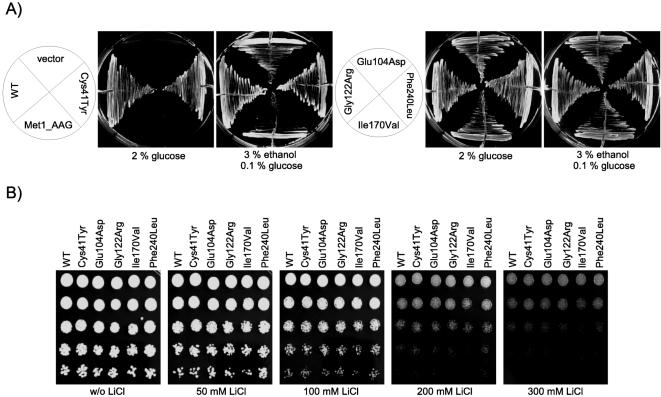
Human wild-type and pathogenic TPI variants can substitute for yeast TPI1. **A)** MR100 yeast cells (*Δtpi1*) were transformed with the various p416GPD-based expression plasmids encoding wild-type human TPI as well as the pathogenic variants Met1_AAG, Cys41Tyr, Glu104Asp, Gly122Arg, Ile170Val or Phe240Leu, respectively, and plated on minimal SC ^-leu-ura^ medium supplemented with 3% ethanol/0.1% glucose. Afterwards, single yeast clones were selected and grown as represented by the schemes on SC ^-leu-ura^ medium plates supplemented either with 2% glucose or with 3% ethanol/0.1% glucose at 30°C. **B)** MR100 *Δtpi1* yeast cells expressing wild-type TPI or the different pathogenic TPI variants were grown until logarithmic phase. Then, the same cell number of each culture was spotted as 5-fold serial dilutions onto glucose media or onto glucose media supplemented with different concentrations of lithium chloride. Plates were incubated for 3 days at 30°C and growth of the different yeast strains was analyzed.

Since it is not feasible to measure the enzymatic activity of one particular TPI enzyme in mammalian cell lines without further endogenous TPI, we decided to exploit yeast as a model system allowing this, since such an approach will give initial insights into potential variations in the enzymatic functionality of the different TPI variants. As described in the Methods section, we have generated the *Δtpi1* strain MR100 deleted for the yeast *TPI1* gene in the haploid BY4741 strain background by a single gene replacement approach. To address the question whether the human wild-type and the pathogenic TPI variants may complement the loss of yeast Tpi1 protein, we transformed this strain with centromeric plasmids for the expression of human wild-type or six representative pathogenic TPI variants as indicated. We observed that yeast cells expressing human wild-type TPI were viable on glucose media, while yeast cells containing the empty vector did not grow on glucose media indicating that the human wild-type enzyme complements the loss of the yeast *TPI1* gene (Fig. 2A). Remarkably, with the exception of the Met1_AAG TPI variant, all pathogenic TPI variants were viable on glucose plates demonstrating functional activity of these enzymes.

Given that a missense mutation in the yeast *TPI1* gene, which eliminates the catalytic activity of the Tpi1 protein, leads to the inhibition of the inositol synthesis pathways resulting in sensitivity to the drug lithium [39], we dropped 5-fold serial dilutions of the *Δtpi1* strain MR100 expressing the various TPI variants on glucose media supplemented with different concentrations of lithium chloride (Fig. 2B). As expected, we did not observe significant growth differences between the various yeast strains even at high concentrations of lithium chloride further confirming glycolytic functionality of the pathogenic TPI variants.

**Figure 3:**
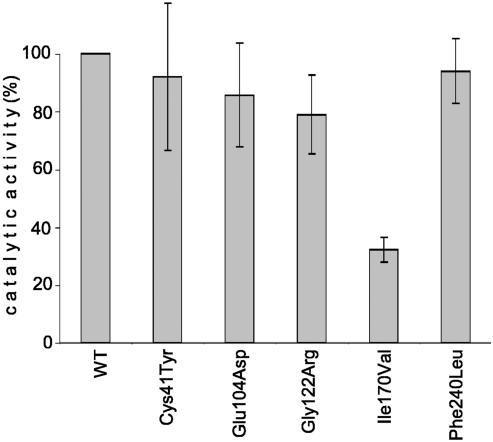
Pathogenic TPI variants display catalytic activity *in vivo*. MR100 *Δtpi1* yeast cells expressing wild-type as well as the pathogenic TPI variants Cys41Tyr, Glu104Asp, Gly122Arg, Ile170Val or Phe240Leu were grown until the logarithmic phase and equal cell numbers were collected. Afterwards, the enzymatic assay was performed as described in the Methods section. The catalytic activity of each pathogenic TPI variant was compared to the activity of the wild-type enzyme. Each column represents the mean value of multiple measurements of 4 different yeast clones. Error bars indicate standard deviation.

Finally, we investigated the catalytic activity of the wild-type and the pathogenic TPI variants in MR100 yeast cells. The *Δtpi1* cells expressing the pathogenic TPI variants Cys41Tyr, Glu104Asp, Gly122Arg or Phe240Leu, respectively, exhibited catalytic activity *in vivo* that was comparable to the catalytic activity of the wild-type protein (Fig. 3). However, yeast cells expressing the Ile170Val TPI variant showed a strong reduction in catalytic activity compared to the wild-type protein and the other pathogenic TPI variants. To rule out that a low intracellular protein level of the Ile170Val TPI variant causes this diminished activity in yeast, we analyzed the expression levels of the different TPI variants in yeast by immunoblot analyses. No differences were detected demonstrating that the decrease in the enzymatic activity of the Ile170Val TPI variant is based on reduced enzymatic activity *per se* (data not shown). Nevertheless, this residual activity of about 30% is sufficient to suppress the growth defect of *Δtpi1* yeast cells on glucose medium as presented in Fig. 2.

### Indications for an alternative translation initiation start site in the TPI mRNA

**Figure 4:**
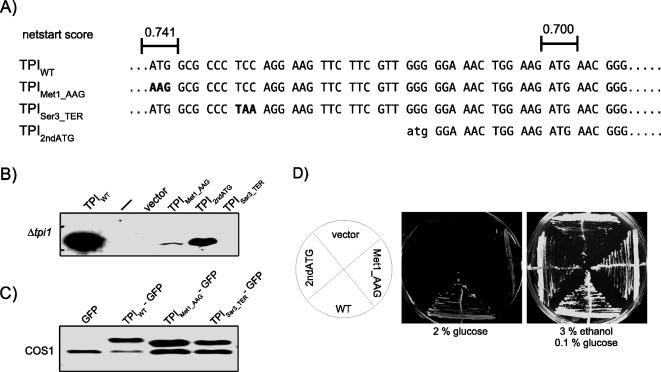
An alternative translation initiation start site within the TPI mRNA. **A)** The first 17 codons of the human TPI sequence and the netstart scores are presented. The introduced mutations are indicated in bold letters. **B)** MR100 *Δtpi1* yeast cells were transformed with the respective plasmids encoding TPI, TPI_Met1_AAG_, TPI_Ser3_TER_ or the variant TPI_2ndATG_. Ethanol lysates were prepared from logarithmically growing yeast cultures and the expression level of the different TPI variants was analyzed by immunoblotting using polyclonal α-TPI serum. Please note that no sample was loaded in case of the lane marked with a dash. **C)** COS1 cells were transfected with the plasmids pEGFP-N1, pEGFP-N1-TPI, pEGFP-N1-TPI_Met1_AAG_ or pEGFP-N1-TPI_Ser3_TER_. Cell lysates were prepared and the expression level of the different fusion proteins was analyzed by immunoblot using an α-GFP antibody. **D)** MR100 *Δtpi1* yeast was transformed with expression plasmids encoding wild-type TPI, TPI_Met1_AAG_ or TPI_2ndATG_. Afterwards, single yeast clones were selected and grown on the respective SC^-leu-ura^ plates for 3 days at 30°C as indicated.

The quite evident discrepancy between patient measurements and *in vitro* data [40] and our findings in yeast clearly support the assumption that other cellular processes are required to cause the extremely low TPI activity measured in patients. On the one hand, a protein's function and activity depends upon the association with its diverse interaction partners occurring in the different cellular compartments. Furthermore, functional diversity of proteins can also be regulated at the translational level due to alternative translation initiation sites within one mRNA transcript probably resulting in protein variants with novel functional properties [41]. Alternative translational start sites occur at a higher frequency within the first 20 codons compared to the frequency in downstream regions [42], [43]. Interestingly, we have noticed that the NetSTART prediction server (http://www.cbs.dtu.dk/services/NetStart), which is based on an algorithm for the prediction of translational start sites [44], gives an exceptionally high confidence score of 0.700 for the ATG codon encoding MET14 within the TPI protein compared to the value of 0.741 for the first start codon (Fig. 4A). Therefore, we analyzed whether the second ATG codon might function as an alternative translational initiation site within the TPI mRNA. To test this, we generated yeast plasmids in which (i) the first ATG codon of the *TPI* gene was replaced by an AAG (as observed in the TPI Paris family), (ii) an artificial stop codon was placed at codon position 3, and plasmids encoding the (iii) wild-type TPI or (iv) TPI_2ndATG_ as presented in Fig. 4A. After transformation of these plasmids into the *Δtpi1* yeast strain, transformants were plated on SC^-ura^ medium supplemented with 3% ethanol/0.1% glucose. Afterwards, ethanol lysates of the different yeast cultures were prepared and the expression level of the different TPI proteins was analyzed by immunoblotting. Wild-type TPI and TPI_2ndATG_ are highly expressed in yeast as shown in Fig. 4B. Interestingly, the TPI variant TPI_Met1_AAG_ was also expressed in yeast demonstrating that the second ATG codon in the *TPI* gene can be used as translational start site. However, no expression of the TPI_Ser3_TER_ variant was observed in yeast, hence, this observation could be based on the high activity of nonsense-mediated mRNA decay in yeast [45]. To confirm the use of the second ATG codon in the *TPI* gene as alternative translational start site in mammalian cell lines, we generated fusion proteins comprising the first 200 nucleotides of human *TPI* gene containing the mutations described above fused to GFP. Afterwards, COS1 cells were transfected with the respective expression plasmids or with the empty plasmid pEGFP-N1 and incubated for two days to allow expression of the recombinant proteins. Cell lysates were prepared and subsequently analyzed by immunoblotting (Fig. 4C). Both fusion proteins TPI_Met1_AAG_-GFP and TPI_Ser3_TER_-GFP were expressed at similar levels compared to the TPI_WT_-GFP protein demonstrating that the second ATG codon within the *TPI* gene can also be used as a translational initiation site in mammalian cells.

To this end, we addressed the question whether the TPI_2ndATG_ variant shows evidence of glycolytic functionality. Again, we set up to exploit our yeast model and transformed *Δtpi1* yeast cells with the plasmid p416GPD-TPI_2ndATG_ to express this variant in yeast. However, yeast cells expressing TPI_2ndATG_ were unable to grow on glucose medium indicating that this protein variant has no catalytic activity (Fig. 4D).

### Dimerization behavior of TPI is affected by the different mutations

**Figure 5:**
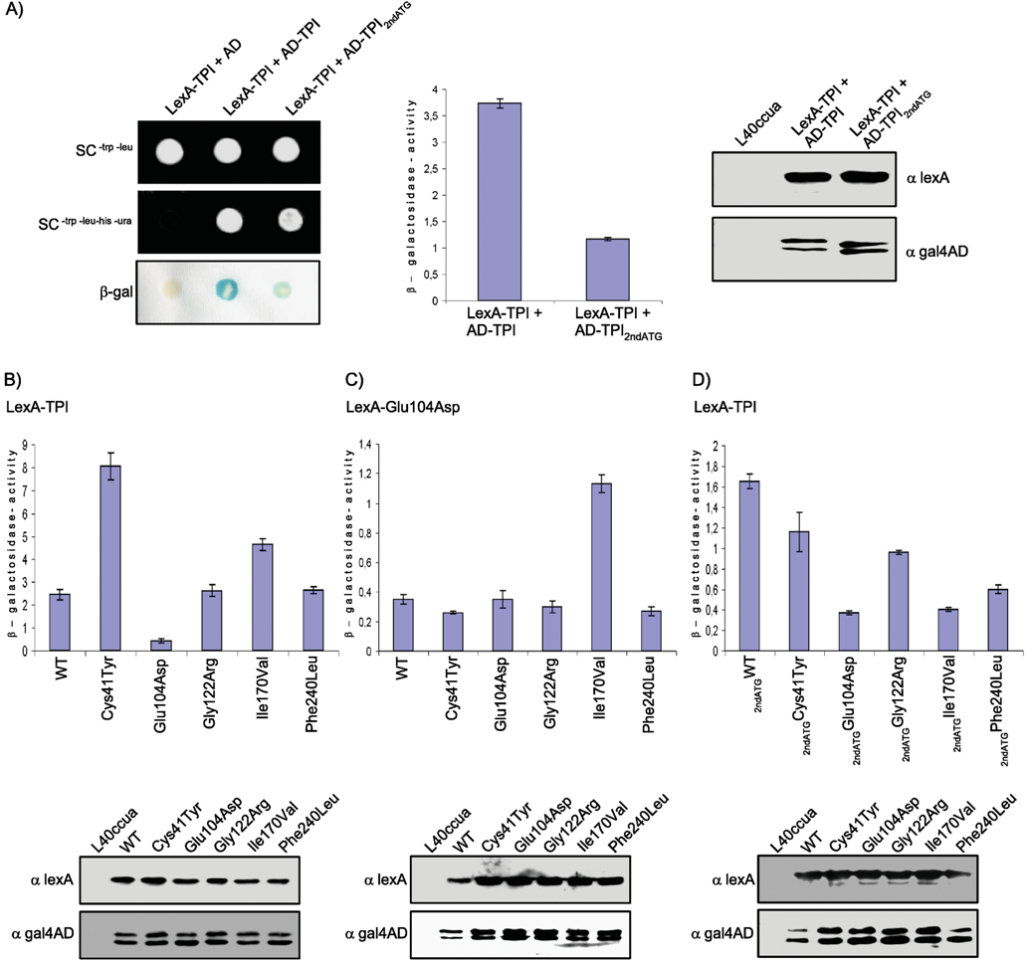
Dimerization behavior of wild-type and pathogenic TPI variants. **A)** The yeast two-hybrid strain L40ccua was co-transformed with plasmids encoding LexA-TPI, AD, AD-TPI or AD-TPI_2ndATG_ as indicated. Afterwards, transformants were spotted on SC media lacking tryptophan and leucine, onto SC medium lacking tryptophan, leucine, histidine and uracil, or onto nylon membrane for analyzing the activity of the three reporter genes (left panel). For quantitative analysis of the *lacZ* reporter gene activity a liquid β-galactosidase assay was performed (middle panel). Each column represents the mean value of four measurements. Error bars indicate the standard deviation. The expression level of the respective bait and prey proteins was analyzed by immunoblot analysis using antibodies against lexA or gal4AD (right panel). **B)** Strain L40ccua was transformed with plasmids encoding LexA-TPI and AD-TPI or LexA-TPI and the pathogenic variants AD-Cys41Tyr, AD-Glu104Asp, AD-Gly122Arg, AD-Ile170Val or AD-Phe240Leu, respectively. In **C)** plasmids encoding the LexA-Glu104Asp protein and AD-WT or LexA-Glu104Asp and the variants AD-Cys41Tyr, AD-Glu104Asp, AD-Gly122Arg, AD-Ile170Val or AD-Phe240Leu, respectively, were used. *LacZ* reporter gene activity in **B)** and **C)** was measured using a liquid β-galactosidase assay. **D)** Dimerization behavior between wild-type TPI and TPI_2ndATG_ variants comprising the corresponding amino acid exchanges was analyzed as described before. All experiments were repeated twice using yeast cells obtained from independent transformations. The intracellular level of the various bait or the prey proteins (in B–D) was monitored by immunoblot analysis using antibodies against lexA or gal4AD.

Abnormal dimerization behavior of the pathogenic TPI variants has often been discussed as a feature in the pathogenesis of TPI deficiency due to the fact that several mutations within the *TPI* gene could influence the dimerization properties of the enzyme [2], e.g., bioinformatic predictions based on domain assignments suggested that the most common mutation at position 104 in the *TPI* gene, which is located in close proximity to the TPI dimerization interface, might affect the dimerization property of TPI ([2], [14] and Fig. 1). However, experimental support for this assumption is lacking. To address this aspect, we exploited the yeast two-hybrid system, since this system allows not only the investigation of the direct interaction between proteins, but also the relative quantification of altered protein interactions [36], [46], [47]. First, we generated constructs encoding the bait fusion protein LexA-TPI or the prey fusion protein AD-TPI to analyze whether dimerization of TPI can be monitored in a directed yeast two-hybrid study and transformed the yeast strain L40ccua with the respective plasmids. Transformants were isolated and the activity of the reporter genes was monitored via growth on SC medium lacking histidine and uracil. We observed that yeast cells co-expressing LexA-TPI and AD-TPI grew on SC^-trp-leu-his-ura^ medium demonstrating activity of the respective reporter genes (Fig. 5A, left panel). In addition, expression of the *lacZ* reporter gene was indicated by a blue color shift in a membrane-based β-galactosidase assay. No activation of the reporter genes was observed in yeast cells expressing the fusion protein LexA-TPI and the AD domain alone demonstrating that complex formation of TPI occurs in the yeast two-hybrid system. Moreover, we analyzed whether wild-type TPI and the TPI_2ndATG_ variant do interact in this system. Yeast cells co-expressing the fusion proteins LexA-TPI and AD-TPI_2ndATG_ exhibited activity of all reporter genes demonstrating that the glycolytic inactive TPI_2ndATG_ variant associates with wild-type TPI. However, the observed β-galactosidase activity resulting from the interaction between wild-type TPI and the TPI_2ndATG_ variant was lower compared to the β-galactosidase activity measured in yeast co-expressing wild-type TPI proteins (Fig. 5A, middle panel). To confirm that the differences in *lacZ* reporter gene activity were not due to different expression levels of the various bait or prey proteins, we analyzed the intracellular levels by immunoblotting and observed no significant differences in intracellular protein concentrations (Fig. 5A, right panel).

In the next step, we investigated whether the mutations within the *TPI* gene might affect the dimerization behavior of the protein by measuring the relative activity of the *lacZ* reporter gene, which indicates the relative strength of a protein-protein interaction [47]. The yeast strain L40ccua was transformed with the bait construct encoding LexA-TPI in combination with prey constructs encoding the TPI variants AD-Cys41Tyr, AD-Glu104Asp, AD-Gly122Arg, AD-Ile170Val or AD-Phe240Leu, respectively. The relative activity of the *lacZ* reporter gene measured in yeast co-expressing LexA-TPI in combination with AD-Gly122Arg or AD-Phe240Leu, respectively, was not significantly altered in comparison to yeast co-expressing LexA-TPI and AD-TPI suggesting that these mutations were not affecting the dimerization behavior with wild-type TPI (Fig. 5B, upper panel). Interestingly, a slightly enhanced β-galactosidase activity was observed in yeast co-expressing LexA-TPI and AD-Ile170Val, whereas a strong enhancement was measured in yeast expressing the fusion proteins LexA-TPI and AD-Cys41Tyr. In addition, the interaction between LexA-TPI and AD-Glu104Asp was greatly reduced as monitored by the strong reduction in the activity of the *lacZ* reporter gene. Again, analysis of the intracellular levels of the bait and prey proteins by immunoblotting demonstrated that all fusion proteins were expressed in yeast at similar levels (Fig. 5B, lower panel). Thus, these results clearly demonstrated that some mutations in the *TPI* gene do indeed lead to an altered dimerization behavior of the TPI enzyme.

Since the Glu104Asp TPI variant is the most common pathogenic TPI variant accounting for approximately 80% of TPI deficiency cases and is the only variant identified to occur in a homozygous state [2], we further explored the interaction properties between this variant and the other pathogenic TPI proteins. We generated a bait plasmid encoding LexA-Glu104Asp and expressed this fusion protein in combination with the various AD fusion proteins as indicated (Fig. 5C). As expected from the results presented in Fig. 5B, the β-galactosidase activity measured in the respective yeast clones was considerably lower compared to the corresponding yeast clones expressing wild-type TPI as bait. Furthermore, no significant alterations in the dimerization properties between the fusion protein LexA-Glu104Asp and the AD-TPI variants Cys41Tyr, Glu104Asp, Gly122Arg, or Phe240Leu, respectively, were detected. Interestingly, yeast co-expressing LexA-Glu104Asp and AD-Ile170Val exhibited a significantly higher β-galactosidase activity. To this end, we also analyzed the dimerization behavior of wild-type TPI and the TPI_2ndATG_ variant carrying the different mutations (Fig. 5D). Yeast co-expressing wild-type TPI and the different TPI_2ndATG_ variants all displayed a reduced dimerization behavior in comparison to yeast cells expressing wild-type TPI and TPI_2ndATG_ proteins. Even here, dimerization was most affected by the Glu104Asp TPI variant. In sum, our results indicate that the different pathogenic mutations highly influence the dimerization behavior of human TPI under recombinant *in vivo* conditions.

### Overexpression of the Glu104Asp TPI variant results in reduced expression levels of endogenous TPI

**Figure 6:**
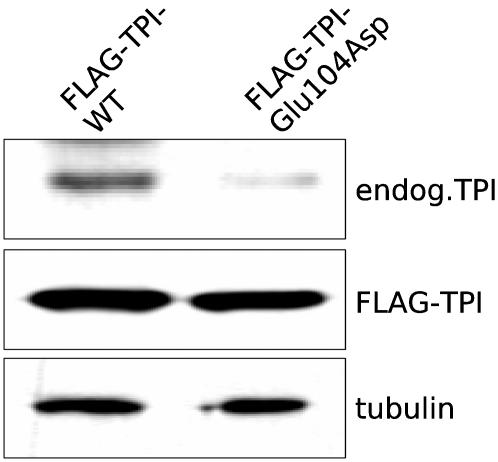
Overexpression of the Glu104Asp TPI variant results in a reduced expression level of endogenous TPI. COS1 cells were transfected with pTL-Flag-TPI-WT or pTL-Flag-TPI-Glu104Asp as indicated. After 48h, lysates were prepared and analyzed by immunoblotting as described in the Methods section. Loading of equal protein concentrations was demonstrated by tubulin staining (lower panel). In addition, similar levels of FLAG-tagged wild-type TPI and FLAG-tagged Glu104Asp TPI was detected in the respective cell lysates (middle panel).

The most frequent TPI variant, Glu104Asp, displays strong defects in the dimerization properties compared to wild-type TPI. This suggests that regulatory processes due to aberrant protein dimerization could contribute to the pathogenesis of TPI deficiency, and therefore we directly investigated whether overexpression of this variant would have an impact on the regulation of endogenous TPI. Therefore, we transfected COS1 cells with plasmids designed for the expression of FLAG-tagged wild-type or pathogenic TPI enzyme. Then, cell lysates were prepared and analyzed by immunoblotting (Fig. 6). Strikingly, in comparison to overexpressed wild-type TPI, a strong reduction in the endogenous TPI level was detected in case the Glu104Asp variant was overexpressed (upper panel). Thus, this result clearly demonstrates that endogenous wild-type TPI is deregulated in the presence of a pathogenic TPI variant exhibiting altered dimerization properties, and hence, one could conclude that this apparent feedback mechanism is potentially accountable for the reduced TPI activity measured in the cell extracts of TPI deficiency patients.

### Reduced TPI activity results in increased cellular resistance against the thiol-oxidizing drug diamide

**Figure 7:**
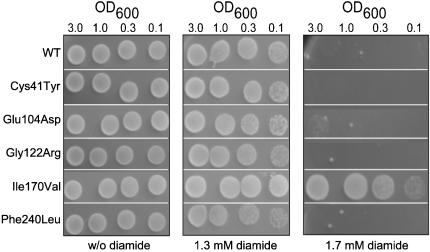
MR100 Δ*tpi1* yeast cells expressing the Ile170Val TPI variant are hyperresistant to diamide. MR100 *Δtpi1* yeast cells expressing wild-type as well as the pathogenic TPI variants Cys41Tyr, Glu104Asp, Gly122Arg, Ile170Val or Phe240Leu were grown to stationary phase, serially diluted to OD_600_ values of 3.0, 1.0, 0.3, 0.1 and spotted onto SC medium plates containing different concentrations of diamide. Sensitivity/resistance was determined by comparing the growth between the different yeast strains after incubating the plates for 3 days at 28°C.

Accumulation of reactive oxygen species (ROS) influences chronological and replicative aging in yeast and plays an important role in the pathogenesis of age related disorders in humans [48]–[50]. Interestingly, increased chronic oxidative stress has been detected in a severely affected TPI deficiency patient as well [20]. In the light of this observation we investigated whether the different pathogenic mutations in the *TPI* gene might cause intracellular accumulation of ROS in exponentially growing *Δtpi1* yeast cultures monitored by dihydroethidium staining. However, no significant differences in red flurorescence between the yeast strains expressing wild-type or the pathogenic TPI variants were observed (data not shown). Finally, we analyzed the resistance of the different yeast strains to oxidants such as hydrogen peroxide, tert-butyl hydroperoxide, cumene hydroperoxide and diamide. No differences in the growth rates between the different yeast strains was monitored on medium containing different concentrations of hydrogen peroxide, tert-butyl hydroperoxide or cumene hydroperoxide, respectively (data not shown). However, we discovered that treatment of the different yeast strains with the thiol-oxidizing drug diamide had an effect. Strikingly, yeast cells expressing Ile170Val TPI, the variant exhibiting a reduced catalytic activity as shown in Fig. 3, were resistant to diamide treatment in comparison to strains expressing wild-type or the other pathogenic TPI variants (Fig. 7). Only yeast cells expressing this TPI variant were able to grow on medium containing 1.7 mM diamide. Thus, this result indicates that a decrease in the catalytic activity of the TPI enzyme mediate cellular resistance against oxidative stress caused by diamide treatment.

## Discussion

Metabolic studies demonstrated that the activity of the TPI enzyme is reduced to 3–20% of the wild-type value as measured in unpurified erythrocyte or muscle extracts of TPI deficiency patients [2], [13], [51]. However, some discrepancies between measurements performed with patients' erythrocytes and a number of observations made in experimental systems and theoretical analyses have been reported over the last few years. As aforementioned, the frequency of *TPI* null alleles is much higher as the rare incidence of TPI deficiency [5]–[9], and *in vitro* measurements of a mutant TPI variant purified from *E. coli* demonstrated that the purified TPI protein carrying the Phe240Leu mutation exhibited a 6-fold higher activity than expected from the measurements performed with the patient erythrocyte extract [40]. Moreover, theoretical calculations demonstrated that the extremely high DHAP level observed in patients' erythrocytes does not correlate with the measured residual TPI activity [13]. Since patients' metabolites highly depend on the genetic background and environmental factors, we have generated an *in vivo* system allowing analysis of the enzymatic activity of wild-type or pathogenic TPI variants inside a cell without additional side effects. Utilizing yeast genetics, we generated a yeast strain deleted for the *TPI1* gene enabling us to investigate the enzymatic and functional properties of a number of human TPI variants *in vivo*. We showed that human wild-type as well as the pathogenic TPI variants Cys41Tyr, Glu104Asp, Gly122Arg, Ile170Val and Phe240Leu, respectively, all suppress the growth defect of *Δtpi1* yeast on glucose medium providing the first *in vivo* evidence that these pathogenic variants exhibit catalytic activity. We corroborated this result by demonstrating that the respective yeast clones are not hypersensitive to lithium chloride, a phenotype caused by inactivation of the Tpi1 enzyme due to inhibition of the inositol biosynthesis pathway [39]. Finally, we provided evidence that the catalytic activity *per se* of all but one pathogenic TPI variant investigated is not affected as a result of the mutation in the *TPI* gene. Interestingly, the pathogenic TPI variant Ile170Val displayed only about 30% of the wild-type catalytic activity; hence, this activity is sufficient to suppress the growth defect of *Δtpi1* yeast on glucose medium.

Since our results seem to be in sharp contrast to the measured TPI activity in unpurified erythrocyte extracts of TPI deficiency patients at disease stage, they clearly make obvious that regulatory events rather than enzyme inactivity are the basis for this enormous reduction in TPI activity. Indeed it has been reported that binding of TPI to an yet unidentified component of red cell membranes can lessen the activity of TPI [23]. Along this line, Jung and co-workers identified human cofilin as interaction partner of TPI and demonstrated that the Rho activator lysophosphatidic acid leads to translocation of cofilin along with TPI to the plasma membrane. This cofilin-TPI complex then interacts with Na/K-ATPase [52]. Consequently, to elucidate the pathogenesis of TPI deficiency, it is of enormous importance to identify cellular factors that interact with TPI, to investigate whether their association has an effect on the catalytic activity of TPI, and obviously whether the binding is affected by the various mutations.

At least three different isoelectric TPI variants encoded by the single *TPI* gene have been observed in human red blood cells [53], [54] or mouse-brain capillary endothelial cells [32]. Furthermore, the Alternative Splicing Database (ASD, [55]) predicts three alternative splice variants for human TPI, but none of them has been identified experimentally to date. Along these lines, protein diversity is often generated by utilizing alternative translation initiation sites [41], [43], mainly if a second start site is located within the first 20 codons [41], [42]. The usage of alternative translation initiation sites have been discussed for disease-causing proteins such as parkin [56], the prion protein [57] and the breast cancer antigen BRCA1 [58]. Here, we provided evidence that the second in-frame ATG codon in the *TPI* gene encoding MET14 can be used as an alternative translation initiation site in yeast as well as in mammalian cells. However, the resulting protein did not suppress the growth defect of *Δtpi1* yeast on glucose medium indicating that this variant lacks catalytic activity. This result is not unexpected, since this TPI variant lacks the catalytic lysine and at least three residues of the intersubunit interface. Interestingly, a TPI deficiency patient which has inherited a start codon mutation in combination with the mutation at position 104, has a stronger pathology in comparison to Glu104Asp homozygotes [17].

Structural alterations of the pathogenic TPI variants have often been speculated to contribute to TPI deficiency as a number of mutations seem to affect the dimerization interface of TPI which forms a stable dimer in most investigated organisms [2], [13], [54]. To address this issue, we have analyzed the dimerization properties between wild-type and the pathogenic TPI variants by a quantitative assay that is based on the *lacZ* gene as reporter. This allows determination of the relative strength of protein-protein interactions in yeast, however, these values can not be directly correlated with binding constants (e.g. K_D_) determined by *in vitro* measurements [46], [47]. We discovered that the dimerization behavior between wild-type TPI and the two pathogenic variants Cys41Tyr and Glu104Asp is strongly altered compared to the dimerization behavior between wild-type proteins. We further observed that the glycolytically inactive TPI_2ndATG_ variant dimerizes with wild-type TPI, although at reduced levels in comparison to the dimerization of wild-type TPI proteins. Finally, we demonstrated that the dimerization between the pathogenic variants also occurred with lower stringency as indicated by the relative activity of the *lacZ* reporter gene. In the light of these results, it is quite remarkable that the Glu104Asp TPI variant, which displayed the most modified dimerization behavior, is the most common variant observed amongst the affected individuals. Moreover, this variant is the only TPI variant found homozygous among affected individuals; all other mutations occur only in the compound heterozygous state together with the Glu104Asp variant or with an obvious *TPI* null allele ([2] and Fig. 1). For example, in the TPI Paris family, the start codon mutant (ATG to AAG) has been detected in the heterozygous state with the Glu104Asp variant [17], the Phe240Leu variant observed in the Hungarian family is found together with the Glu145TER null allele [18]. Generally, dimerization between proteins is a substantial process to generate and regulate cellular functions of proteins [59]. Remarkably, we discovered that overexpression of the Glu104Asp variant results in reduced levels of endogenous TPI in mammalian cells. This finding is exciting since recent studies demonstrated that both compound heterozygous brothers of the Hungarian family have reduced TPI mRNA levels compared to their parents and healthy controls [60] suggesting that altered dimer formation resulting in a modified feedback regulation of the TPI enzyme could underlie the pathogenesis of TPI deficiency.

Remarkably, TPI is highly up-regulated under stress conditions in mammalian cells ([32] and Ralser, unpublished observations). Regarding this issue, it is important that expression of the Ile170Val TPI variant with reduced catalytic activity results in higher resistance of yeast to diamide. Diamide is an oxidant which is known to stoichiometrically oxidize small molecules including glutathione (GSH) within red blood cells, but is less effective in oxidizing protein-integrated cysteines [61]. Therefore, diamide treatment results in a rapid decrease in intracellular GSH, thus, leading to oxidative stress [62]. Interestingly, the response mechanisms of eukaryotic cells to oxidative stress are strictly dependent on the compound and its downstream effects [63]. This might explain why yeast cells expressing the TPI variant with reduced catalytic activity were not resistant to all oxidants examined. It is noteworthy to mention that in contrast to diamide, all other oxidants used in this study are hydroperoxides. Treatment with hydroperoxides leads to the inactivation of the Tdh3 protein, the most abundant of the three glyceraldehyde-3-phosphate dehydrogenase (GAPDH) enzymes in yeast, by S-thiolation, carbonlyation or ADP-ribosylation [64]–[67]. GAPDH catalyzes the first downstream reaction after TPI in glycolysis and remarkably, mutants lacking *TDH3* were sensitive to a challenge with a lethal dose of H_2_O_2_ [67]. It is likely that inactivation of GAPDH after peroxide treatment of yeast cells is forestalling the protective effect of TPI variants exhibiting reduced catalytic activity. In this context it is noteworthy to mention that blockage of glycolysis can force an increased influx of metabolites into the pentose phosphate pathway resulting in an elevated cellular NADPH concentration [68], [69] and vice-versa that different mutations introduced in enzymes implicated in this pathway are leading to oxidant-hypersensitive cells [70]. High intracellular NADPH levels are beneficial during conditions of oxidative stress, because NADPH provides the base for several antioxidant enzymes including the thioredoxins or the glutaredoxin system [71], [72]. As aforementioned, the first downstream enzyme of TPI in glycolysis, GAPDH, is specifically inactivated after peroxide treatment of yeast cells [64], [66] and this subject is, interestingly, reflected in mammalian cells as well [65], [68].

**Figure 8:**
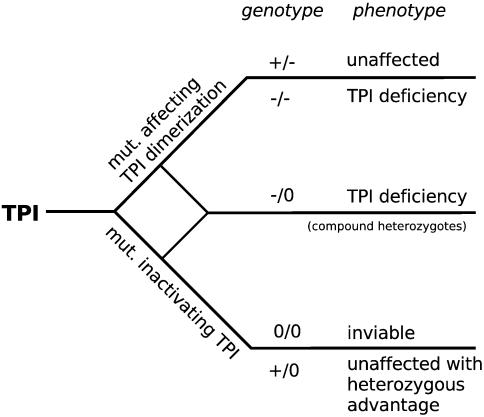
Consequences of mutations within human TPI. Homozygous mutations within TPI affecting enzyme dimerization cause TPI deficiency, whereas homozygous mutations resulting in an inactive *TPI* allele are lethal. Compound heterozygous individuals having inherited one inactive and one allele defective in dimerization properties will develop TPI deficiency, whereas heterozygote individuals having inherited a heterozygote null allele have an evolutionary advantage. (+: wild-type TPI; −: TPI with aberrant dimerization property; 0: allele encoding no or a catalytically inactive TPI).

In the light of the above-mentioned findings it is quite intriguing that the frequency of heterozygous individuals carrying one inactive *TPI* allele is quite high. Several studies demonstrated an allelic frequency from roughly 0.002 (Caucasian and Japanese population) to 0.02 (African American population) [5]–[9]. Indeed this number implies that 1 out of 2000 newborn individuals from the latter population would suffer from this tremendous disorder, but less than 100 individuals have been diagnosed with TPI deficiency worldwide [6]. A mutagenesis screen in mice identified four heterozygous TPI mutations that lead to a 50% reduction in catalytic TPI activity in several tissues examined [12]. However, each homozygous or compound heterozygous offspring of these mice, which lacked any detectable phenotypical abnormalities in the heterozygote state, resulted in early embryonic lethality [12]. These results demonstrated that mutations in the *TPI* gene resulting in a catalytic inactive TPI enzyme cause homozygote embryonic lethality in mice. The human population studies performed are indicating that this situation is reflected in humans as well [5], [6]. Consequently, the occurring mutations in *TPI* alleles of TPI deficiency patients cannot encode catalytically inactive TPI enzymes. As illustrated and demonstrated in Fig. 8, homozygote *TPI* null alleles are lethal, since no homozygotes carrying *TPI* null alleles can be detected in mouse and humans [5], [12]. Furthermore, TPI variants with altered dimerization properties and exhibiting almost normal specific catalytic activity like the Glu104Asp variant, occur in homozygous states causing TPI deficiency [14] or in combination with *TPI* null alleles, for instance, the TPI Paris (Met1_AAG; heterozygous with Glu104Asp) or TPI Alfortwille (frameshift at codon 29; heterozygous with Glu104Asp) [17] representing the compound heterozygote state, whereas heterozygote individuals having inherited a heterozygote null allele in combination with the wild-type allele are healthy and might have a heterozygous advantage. Regarding this issue, one should keep in mind that advantage of heterozygous state has been demonstrated for sickle cell anemia and cystic fibrosis, two other genetic disorders. Strikingly, the allelic frequency of mutations causing cystic fibrosis is comparable to the frequency of heterozygous TPI mutations in the examined Afro-American population [6], [73]. The fact that the yeast strain harboring the TPI variant with reduced catalytic activity is more resistant to specific oxidative stress is a first indication that mutations within *TPI* alleles resulting in catalytically impaired enzymes could confer an advantage to certain stress stimuli or other environmental conditions.

## Supporting Information

Primer Sequences and Plasmids (0.06 MB DOC)
