# Supplementary material for: Triose Phosphate Isomerase Deficiency Is Caused by Altered Dimerization–Not Catalytic Inactivity–of the Mutant Enzymes
Source: PLoS One. 2006 Dec 20;1(1):e30. doi: 10.1371/journal.pone.0000030 (PMC1762313; doi:10.1371/journal.pone.0000030)
Supplement: Primer Sequences and Plasmids — (0.06 MB DOC) [file pone.0000030.s001.doc]

Supporting Information for

**Triose Phosphate Isomerase Deficiency is caused by altered dimerization – not catalytic inactivity – of the mutant enzymes.**

Markus Ralser, Gino Heeren, Michael Breitenbach, Hans Lehrach and Sylvia Krobitsch

**Table S1: Plasmids used in this study**

| ***Name*** | ***Acc. No.*** | ***Ref.*** | ***description*** |
| --- | --- | --- | --- |
| p416GPD | DQ269148 | [1] | Yeast centromeric expression vector with *GPD* promoter and *URA3* marker |
| p426GPD | DQ019861 | [1] | Yeast 2µ expression vector with *GPD* promoter and *URA3* marker |
| pEGFP-N1 | U55762 | Clontech | Mammalian expression vector with CMV promoter for expression of C-terminal GFP-fusion proteins |
| pTL-FlagC | *n.a.* |  | Mammalian expression vector based on the pSG5 backbone [2] with SV40-early promoter for overexpression of FLAG-tagged proteins |
| pBTM117c | *n.a.* | [3] | Yeast two-hybrid vector for expression of  LexA-fusion proteins, based on pBTM116 |
| pACT4-1b | *n.a.* | [4] | Yeast two-hybrid vector for expression of AD-fusion proteins, based on pACT2 (Clontech) |

**Table S2: Oligonucleotides used to generate the yeast strain MR100 (Δ*tpi1*)**

| Name | Sequence |
| --- | --- |
| *LEU2::tpi1*-s | tctataactacaaaaaacacatacataaactaaaa**ATG**TCTGCCCCTAAGAAGAT |
| *LEU2::tpi1*-as | aaagaagataatatttttatataattatattaatc**TTA**AGCAAGGATTTTCTTAA |
| *TPI1*-locus-s | TAATTAAAGCAATCACACAATTCTCTCGG |
| *TPI1*-locus-as | GTGGCTCAGAATGAAAAAGAAACAA |

The oligonucleotides *TPI1*-locus-s and *TPI1*-locus-as were used for genomic amplification of the yeast *TPI1* locus.

**Table S3: Oligonucleotides used to create TPI variants Cys41Tyr, Glu104Asp, Gly122Arg, Ile170 Val and Phe240Leu**

| TPI-Cys41Tyr | s accgaggtggtttatgctccccctactgcc  as ggcagtagggggagcataaaccacctcggt |
| --- | --- |
| TPI-Glu104Asp | s catgtctttggggactcagatgagctgatt  as aatcagctcatctgagtccccaaagacatg |
| TPI-Gly122Arg | s gcagagggactccgagtaatcgcctgcatt  as aatgcaggcgattactcggagtccctctgc |
| TPI-Ile170 Val | s gagcctgtgtgggccgttggtactggcaag  as cttgccagtaccaacggcccacacaggctc |
| TPI-Phe240Leu-as-Not1 | TATAGCGGCCGC**tca**ttgtttggcattgatgatgtccacgagttc |

**Table S4: Oligonucleotides used to create expression plasmids p416GPD-TPI and p426GPD-TPI**

| Name | Sequence |
| --- | --- |
| TPI-s-BamH1 | GAGGATCC**ATG**GCGCCCTCCAGGAAGTT |
| TPI_2ndATG_-s-BamH1 | GAGGATCCATGGGAAACTGGAAG**ATG**AACGG |
| TPI-as-Xho1 | GAGCTCGAG**TCA**TTGTTTGGCATTGATGA |

**Table S5: Oligonucleotides used to introduce start codon mutations or artificial stop codons**

| Name | Sequence |
| --- | --- |
| TPIMet1_AAG-s-BamH1 | GAGGATCC**AAG**GCGCCCTCCAGGAAGTTCTTCGTTGGG |
| TPISer3_TER-s-BamH1 | GAGGATCC**ATG**GCGCCC**TAA**AGGAAGTTCTTCGTTGGG |
| TPI-pEGFPN1-s-Sal1 | GATGTCGACA**ATG**GCGCCCTCCAGGAAGTT |
| TPIMet1_AAG-pEGFPN1-s-Sal1 | GATGTCGACA**AAG**GCGCCCTCCAGGAAGTT |
| TPISer3_TER-pEGFPN1-s-Sal1 | GATGTCGACA**ATG**GCGCCC**TAA**AGGAAGTT |
| TPI-pEGFPN1-as-BamH1 | CTGGATCCCAGTTCTGCGCAGCCA |

**Table S6: Oligonucleotides used to create yeast two-hybrid plasmids**

| Name | Sequence |
| --- | --- |
| TPI-s-Sal1 | GCGTCGACG**ATG**GCGCCCTCCAGGAAGTT |
| TPI-as-Not1 | CATAGCGGCCGC**TCA**TTGTTTGGCATTGATGA |
| TPI_2ndATG_-s-Sal1 | GAGTCGACAGGAAACTGGAAG**ATG**AACGG |

All nucleotide sequences are aligned in 5` - 3`direction and underlined sequences represent restriction sites. Start and Stop codons are highlighted. PCR reactions were performed using Vent® proofreading polymerase. All DNA modifying and restriction enzymes were obtained from New England Biolabs and used according to the manufacturers recommendations.

**References**

1. Mumberg D, Muller R, Funk M (1995) Yeast vectors for the controlled expression of heterologous proteins in different genetic backgrounds. Gene 156: 119-122.

2. Green S, Issemann I, Sheer E (1988) A versatile in vivo and in vitro eukaryotic expression vector for protein engineering. Nucleic Acids Res 16: 369.

3. Goehler H, Lalowski M, Stelzl U, Waelter S, Stroedicke M, et al. (2004) A protein interaction network links GIT1, an enhancer of huntingtin aggregation, to Huntington's disease. Mol Cell 15: 853-865.

4. Stelzl U, Worm U, Lalowski M, Haenig C, Brembeck FH, et al. (2005) A human protein-protein interaction network: a resource for annotating the proteome. Cell 122: 957-968.
